# Hemimegalencephaly: Evolution From an Atypical Focal Early Appearance on Fetal MRI to More Conventional MR Findings

**DOI:** 10.7759/cureus.27976

**Published:** 2022-08-13

**Authors:** David Kakish, Marie Tominna, Anant Krishnan

**Affiliations:** 1 Department of Radiological Sciences, University of California, Irvine, Irvine, USA; 2 Department of Diagnostic Radiology & Molecular Imaging, Beaumont Hospital, Oakland University William Beaumont School of Medicine, Royal Oak, USA

**Keywords:** hemimegalencephaly, pediatrics & neonatology, fetal imaging, pediatric radiology, neonatal neurology, congenital birth defect, congenital malformation, neuroradiology, focal hemimegalencephaly, unilateral megalencephaly

## Abstract

Hemimegalencephaly, or unilateral megalencephaly, is a sporadic congenital brain malformation characterized by enlargement of a cerebral hemisphere due to an abnormal proliferation of neurons or glial cells. Hemimegalencephaly is part of a spectrum of disorders, increasingly referred to as mTORopathies, which arise as a result of dysregulation or hyperactivation of the mammalian target of rapamycin (mTOR)-signaling cascade resulting in less restricted cell growth and survival. The resultant cortical disorganization and enhanced neuronal excitability often manifest clinically in the form of seizures. Ultrasound and magnetic resonance imaging (MRI) are often used to characterize hemimegalencephaly. Typical imaging findings seen include diffuse unilateral enlargement of a cerebral hemisphere with overlying cortical malformation and ipsilateral dilation of the lateral ventricle.

This paper will review an unusual case of focal hemimegalencephaly diagnosed on prenatal imaging. Initial *in utero* MRI revealed a mass-like lesion in the frontal lobe without associated perilesional cerebral edema. Keying in on abnormalities within the overlying cortex was crucial in suggesting focal hemimegalencephaly as a leading diagnosis and distinguishing it from alternative diagnoses such as a neoplasm. Follow-up fetal MRI demonstrated the evolution of the cerebral abnormality and confirmed the diagnosis. Early diagnosis facilitated appropriate counseling of the parents and guided postnatal imaging and management.

## Introduction

Hemimegalencephaly is a rare congenital hamartomatous brain malformation with diffuse or focal enlargement of a cerebral hemisphere as a result of neuronal or glial proliferation [1,2]. It is believed to occur as a result of de novo mutations in somatic genes involved in the regulation of the mammalian target of rapamycin (mTOR)-signaling cascade; subsequently, this spectrum of diseases is labeled mTORopathies [1,2]. In the case of hemimegalencephaly, the most common gene involved is PIK3CA [1]. Since the mTOR pathway is involved in the regulation of cell growth and death, enhanced activation of this pathway due to genetic mutations propagates this abnormal development [1,2]. It is believed that the timing of the mutation during neurogenesis determines the pathology such that widespread and early mutations may result in hemimegalencephaly while more localized and delayed mutations may produce focal cortical dysplasia, a less extensive congenital abnormality also under the mTORopathy label [1,3]. The resultant structural disorganization, along with the enhanced neuronal excitability that may be associated with mTOR hyperactivation, is often associated with intractable seizures [1-4]. In addition to epilepsy, patients may present with macrocephaly, developmental delay, intellectual disability, and paralysis [1,5].

The involved hemisphere may have focal or diffuse neuronal migration defects, enlargement of the ipsilateral lateral ventricle, white matter gliosis, as well as an abnormal gyral pattern such as agyria, pachygyria, polygyria, and polymicrogyria [1,6-8]. In some cases, the ipsilateral brainstem and cerebellum may be hypertrophic in addition to the cerebral hemisphere, a phenomenon known as total hemimegalencephaly [1,7].

Hemimegalencephaly is a sporadic disorder. The prevalence is distributed equally by sex and is believed to be 1-3 per 1,000 epileptic children [1,9]. It is an isolated finding in the majority of cases, but may also be associated with neurocutaneous syndromes, including epidermal nevus syndrome, proteus syndrome, hypomelanosis of Ito, neurofibromatosis type I, Klippel-Trenaunay syndrome, and tuberous sclerosis [1,2,4].

In this report, we review the prenatal imaging findings of focal hemimegalencephaly, a variant of hemimegalencephaly in which there is focal enlargement within a cerebral hemisphere.

## Case presentation

A 37-year-old primigravida presented for routine prenatal care and underwent a fetal ultrasound at 25 weeks’ gestation. The prenatal ultrasound demonstrated an absent cavum pellucidum with midline shift. There was a suggestion of possible enlargement of the right frontal lobe. Fetal magnetic resonance imaging (MRI) was recommended for further characterization of the abnormalities.

**Figure 1:**
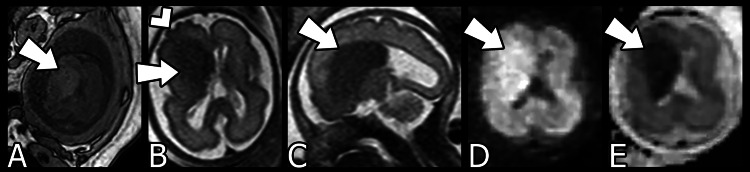
Fetal MRI at 26 weeks’ gestation A) Axial T1-weighted, B) Axial T2-weighted, C) Sagittal T2-weighted, D) Diffusion-weighted imaging (DWI), E) Apparent diffusion coefficient (ADC). The arrows demonstrate a mass-like focus in the region of the right frontal germinal matrix with increased signal on T1-weighted imaging and decreased signal on T2-weighted imaging. Additionally, there is associated restricted diffusion as evidenced by increased signal on DWI and decreased signal on ADC. The arrowhead indicates cortical irregularity along the right frontal lobe suspicious for polymicrogyria.

A fetal MRI performed at 26 weeks’ gestation (Figure 1) revealed a hypointense mass-like focus in the region of the right frontal germinal matrix, or ganglionic eminence, with an enlarged right frontal lobe. Cortical irregularity of the right frontal lobe was also suspected and felt to be concerning for polymicrogyria. Right colpocephaly was also noted but a definite occipital cortical abnormality was not seen. This constellation of findings was concerning for a lobar or partial hemimegalencephaly versus a large area of cortical dysplasia.

The patient and her husband were counseled at a multidisciplinary conference with a maternal fetal medicine specialist, neuroradiologist, and pediatric neurologist. Given the fairly focal abnormality, concern was raised for other pathologies; however, hemimegalencephaly was the leading concern. A follow-up fetal MRI to reevaluate was offered and the patient agreed.

**Figure 2:**
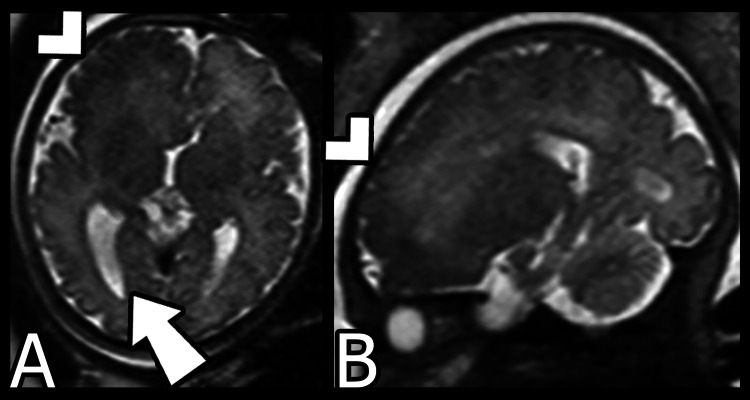
Fetal MRI at 36 weeks’ gestation A) Axial T2-weighted, B) Sagittal T2-weighted. The previously focal abnormality has evolved into a more diffusely dysplastic appearance of the right frontal lobe. The arrowheads indicate an area of cortical thickening and irregular gyration which suggest diffuse cortical malformation. The arrow demonstrates a distinctly enlarged right occipital horn.

Subsequent fetal MRI performed at 36 weeks’ gestation (Figure 2) demonstrated evolution of the focal abnormality into a more diffuse right frontal dysplastic appearance. There was persistent asymmetric enlargement of the right frontal lobe with areas of cortical thickening and irregular gyration. An enlarged right occipital horn was also noted. These findings were now more typical of hemimegalencephaly. While the previously observed central mass-like lesion was no longer observed, the basal ganglia was poorly developed and there was a compressed frontal horn.

**Figure 3:**
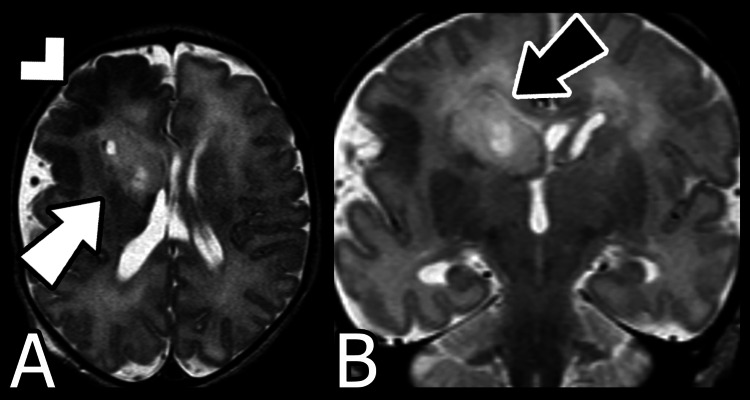
Postnatal MRI on day 3 A) Axial T2-weighted, B) Coronal T2-weighted. The arrowhead emphasizes the severe dysplasia of the right frontal cortex. The white arrow demonstrates dysmorphia of the right basal ganglia and periventricular region with cystic cavities. The black arrow denotes a pointed right frontal horn.

The infant was born at term by Caesarian section. Postpartum physical examination demonstrated mild left hemiparesis. MRI on day three of life (Figure 3) revealed a more diffusely enlarged right cerebral hemisphere with a dysplastic right frontal lobe. The right basal ganglia and periventricular region were dysmorphic with cystic cavities as well as possible calcifications. A dilated right occipital horn was seen again and a pointed right frontal horn became more apparent.

An electroencephalogram (EEG) revealed multifocal right hemispheric epileptiform discharges and right frontocentral seizures which were either subclinical or possibly subtly clinical with left upper extremity waving and left hemibody stiffening. He was immediately started on levetiracetam, followed by phenobarbital, and then topiramate before seizure control was achieved.

At the age of two months, he developed infantile spasms which were treated with adrenocorticotropic hormone (ACTH) and ultimately vigabatrin. Over the next few months, the patient was successfully weaned off phenobarbital but otherwise remained on the complex antiseizure regimen.

Ultimately, the infant underwent a right hemispherectomy at eight months of age. Anatomic pathology results were consistent with a cortical developmental malformation. His remaining antiepileptic medications were gradually weaned off over the following year. The infant has remained clinically seizure-free since the surgery.

## Discussion

This report illustrates a case of hemimegalencephaly that presented as a focal variant during the second trimester of pregnancy. Findings on prenatal ultrasound that should raise suspicion for hemimegalencephaly include unilateral ventriculomegaly, asymmetric cerebral hemispheres with midline shift in the occipital region, subtle irregular sulcation or cortical thickening, and increased head circumference in the 90th percentile [1,8,10].

On MRI, the abnormality was characterized by low signal on T2-weighted imaging which was likely a result of the high cellularity in the malformed region [10]. The unusual initial mass-like appearance with central T2 hypointensity was potentially a reflection of both the early prenatal timing of the initial MRI and the focality of involvement which may have been due to hyperplasia of the ventricular zone and germinal matrix. Over time, the imaging findings evolved into the more classical appearance of hemimegalencephaly as characterized by more diffuse cerebral hemispheric enlargement, more pronounced abnormal cortical gyri, and more apparent dilation of the occipital horn of the ipsilateral lateral ventricle.

The diagnosis of focal hemimegalencephaly can be difficult to make based on prenatal ultrasound alone. If there is evidence of unilateral ventriculomegaly on ultrasound, in utero MRI should be considered [10]. On MRI, the cerebral cortex may appear unremarkable or dysplastic; specifically, a dysplastic cortex may exhibit polymicrogyria, lissencephaly, agyria, pachygyria, or gray matter heterotopia. Due to the cortical heterotopia and astrocytosis, MRI often demonstrates an indistinct junction between the affected cortex and the adjacent white matter [7]. Additionally, MRI may demonstrate a dilated lateral ventricle with a straight and anterosuperiorly pointed frontal horn [7,8].

Other diagnoses for a mass-like lesion must also be considered on the differential. Specifically, a true mass or neoplasm may exhibit a similar appearance on T2-weighted MRI. Subtle overlying cortical irregularities on prenatal ultrasound and MRI can suggest the diagnosis of focal hemimegalencephaly over a true mass or neoplasm. Additionally, while a neoplasm would be expected to demonstrate mass effect as well as peritumoral cerebral edema, focal hemimegalencephaly may not [8,11]. Finally, repeat imaging over the course of the pregnancy may assist with narrowing the differential diagnoses [11]. In our case, follow-up MRI demonstrated evolution of the mass-like lesion and more conventional radiologic findings for hemimegalencephaly later in the pregnancy. Postpartum imaging reinforced the diagnosis of hemimegalencephaly and allowed for definitive management.

It is important to have an understanding of the early prenatal appearance of the focal form of hemimegalencephaly to facilitate appropriate care of the mother and infant. Early suspicion and diagnosis on fetal MRI allowed for appropriate counseling and discussions with the parents, set expectations regarding potential outcomes including the risk of intractable seizures, and allowed for a planned delivery with postpartum neonatal EEG monitoring. Thus, timely radiologic diagnosis of hemimegalencephaly expedited management and subsequently reduced overall seizure burden, ultimately minimizing developmental abnormalities and optimizing long-term outcomes [12-14].

## Conclusions

This case is unusual due to the initial low signal on T2-weighted sequences and the central, mass-like appearance of the right frontal germinal matrix with a lack of more diffuse unilateral features that are often described and expected in hemimegalencephaly. However, subsequent prenatal imaging demonstrated evolution into more typical radiologic findings associated with hemimegalencephaly which was confirmed on postnatal imaging. For prenatal imagers, it is vital to recognize the potential initial appearance of early focal dysplastic hemimegalencephaly as an awareness of this prospective presentation ultimately allows for and facilitates appropriate maternal and neonatal care.
